# The Medical Curriculum

**Published:** 1902-09-06

**Authors:** 


### The Medical Curriculum.

Although there are differences in detail between the courses of study fixed by the various qualifying bodies for those who aspire to these diplomas or degrees as the case may be, there is of necessity a general similarity among them seeing that the regulations of all these bodies must conform to those laid down by the General Medical Council. Whatever qualification the student seeks he must pass five years in study after he has been registered as a medical student.

Before he can be registered as a medical student, *i.e.*, before his medical studies will be allowed to "count," he must have passed one of the recognised Preliminary Examinations in Arts recognised by the General Medical Council, a complete list of which can be obtained from the Registrar of the General Medical Council, 299 Oxford Street, London, W. In making his selection from this long list it must be borne in mind that the matriculation examination at some of the Universities is itself accepted as a Preliminary in Arts, so that when it has been passed no other Preliminary is required. On the other hand it must not be forgotten that in the case of a student seeking a university degree, the particular Preliminary Examination demanded by the university must be passed, or else the whole thing will have to be done over again.

After registration the course of medical study may be roughly divided into three very distinct periods, each devoted to a certain class of work and each ending with an examination by which the work is tested. As a general rule the whole of the student's first year is devoted to the subjects of the First Examination which is usually passed in three parts—(1) Biology, at the end of the first winter session; (2) Chemistry and Physics at the end of the summer session in July; and (3) Pharmacy, which may be passed during the first year or may be postponed. It is a great comfort, however, to get it over at its proper time.

The second division of the curriculum is devoted to the study of the intermediate medical subjects, namely, Anatomy and Physiology. The earliest time at which the student can be admitted to the Second Examination is twelve months after completing the First Examination, or if he has passed the First Examination before entering at a medical school (a course which is permissible) after two winter and one summer session of attendance at a recognised medical school.

Having passed the Second Examination the student begins systematic work in the wards, the operating theatres, the out-patient department, and the dead-house, in all of which, however, he will, if he be well advised, have already spent a not inconsiderable number of odd hours.

The Third or Final Examination is passed at the end of five years from the date of registration, and at the expiration of four years after the date of passing the First Examination, and of two years from the date of passing the Second Examination. It is the fixity of these dates, and the necessity of interpolating between the several examinations certain definite periods of study, that makes "plucking" at an examination so serious a matter, for the time so lost cannot be made good.

